# Design and characterization of genetically engineered zebrafish aquaporin-3 mutants highly permeable to the cryoprotectant ethylene glycol

**DOI:** 10.1186/1472-6750-11-34

**Published:** 2011-04-08

**Authors:** François Chauvigné, Esther Lubzens, Joan Cerdà

**Affiliations:** 1 Laboratory of Institut de Recerca i Tecnologia Agroalimentàries (IRTA)-Institut de Ciències del Mar, Consejo Superior de Investigaciones Científicas (CSIC), 08003 Barcelona, Spain; 2 Department of Marine Biology, Israel Oceanographic and Limnological Research, 81080 Haifa, Israel

## Abstract

**Background:**

Increasing cell membrane permeability to water and cryoprotectants is critical for the successful cryopreservation of cells with large volumes. Artificial expression of water-selective aquaporins or aquaglyceroporins (GLPs), such as mammalian aquaporin-3 (AQP3), enhances cell permeability to water and cryoprotectants, but it is known that AQP3-mediated water and solute permeation is limited and pH dependent. To exploit further the possibilities of using aquaporins in cryobiology, we investigated the functional properties of zebrafish (*Danio rerio*) GLPs.

**Results:**

Water, glycerol, propylene glycol and ethylene glycol permeability of zebrafish Aqp3a, -3b, -7, -9a, -9b, -10a and -10b, and human AQP3, was examined. Expression in *Xenopus laevis *oocytes indicated that the permeability of DrAqp3a and -3b to ethylene glycol was higher than for glycerol or propylene glycol under isotonic conditions, unlike other zebrafish GLPs and human AQP3, which were more permeable to glycerol. In addition, dose-response experiments and radiolabeled ethylene glycol uptake assays suggested that oocytes expressing DrAqp3b were permeated by this cryoprotectant more efficiently than those expressing AQP3. Water and ethylene glycol transport through DrAqp3a and -3b were, however, highest at pH 8.5 and completely abolished at pH 6.0. Point mutations in the DrAqp3b amino acid sequence rendered two constructs, DrAqp3b-T85A showing higher water and ethylene glycol permeability at neutral and alkaline pH, and DrAqp3b-H53A/G54H/T85A, no longer inhibited at acidic pH but less permeable than the wild type. Finally, calculation of permeability coefficients for ethylene glycol under concentration gradients confirmed that the two DrAqp3b mutants were more permeable than wild-type DrAqp3b and/or AQP3 at neutral pH, resulting in a 2.6- to 4-fold increase in the oocyte intracellular concentration of ethylene glycol.

**Conclusion:**

By single or triple point mutations in the DrAqp3b amino acid sequence, we constructed one mutant with enhanced ethylene glycol permeability and another with reduced pH sensitivity. The DrAqp3b and the two mutant constructs may be useful for application in cryobiology.

## Background

Over the last decades, advances in understanding basic phenomena in cryobiology has led the development of effective methods for the preservation of a very wide range of cells [1]. However, the cryopreservation of cells with large volumes and multicellular systems and tissues, such as vertebrate oocytes and embryos, still remains a challenge in some cases [2-4]. Vertebrate oocytes show low surface area to volume ratio and low permeability to water and cryoprotectants, and as a consequence they are highly susceptible to formation of intracellular ice, cryoprotectant-toxicity, and osmotic stress. In addition, oocyte cryosurvival may change during development or as a result of different genotypes [4,5].

One of the major obstacles with freezing of a cell is that intracellular water content crystallizes, leading to mechanical and physical damage [6,7]. To limit the rate of ice crystals formation, the first step is to permeate cells with cryoprotectant agents, such as glycerol, propylene glycol, dimethyl sulfoxide or ethylene glycol, which should reach high intracellular concentrations, thus generating the driving force for the efflux of water by osmosis and of solutes by diffusion [6,7]. This can be achieved either by a slow cooling rates with low concentration of cryoprotectants, or fast cooling after incubation in highly concentrated cryoprotectant solutions (vitrification) [8,9]. An ideal cryoprotectant should thus exhibit low toxicity and rapidly permeate the cell, so that osmotic volume changes, that are a recognized form of cellular stress, are minimized [10-12]. Ethylene glycol shows the best efficiency/toxicity ratio on mammalian oocytes and embryos and is thus the most common selected cryoprotectant for slow-cooling or vitrification protocols [13-18]. However, the permeability of vertebrate oocytes and embryos to ethylene glycol is limited [19-24] and may change during development and differentiation [3,4], resulting possibly in intracellular damage after cryopreservation [4,16,25].

It is now well established that biological membranes allow water to pass by simple diffusion through the lipid bilayer, or when rapid water permeability is required (in case of reabsorption, secretion or osmotic stress), through specialized membrane channels known as aquaporins. Aquaporins (AQPs) are integral membrane proteins present in all living organisms from bacteria to mammals [26]. These proteins consist of six transmembrane helices connected by five loops, two of which bear the highly conserved asparagine-proline-alanine (NPA) motifs involved in the formation of the water pore [26]. To date, 13 aquaporins have been identified in mammals (AQP0-12) and while most of them are water-selective, functional studies have identified a subgroup of channels (AQP3, -7, -9 and -10) that can transport also glycerol and urea, and are termed aquaglyceroporins (GLPs) [26,27]. Some GLPs are also permeated by metalloids and a wide variety of noncharged solutes (e.g., carbamides, polyols, purines, pyrimidines), including cryoprotectants such as propylene glycol, ethylene glycol, acetamide and possibly dimethyl sulfoxide [28-32].

The discovery of aquaporins made it possible to use them for enhancing water and cryoprotectant permeability of cells [33-35]. In the baker's yeast (*Saccharomyces cerevisiae*), freeze tolerance during rapid freezing conditions positively correlates with high levels of expression of the water-selective aquaporins Aqy1 and Aqy2 [33,36]. This positive effect has been explained by the rapid water efflux, especially at freezing temperatures, as a consequence of high levels of aquaporins in the plasma membrane of yeast cells, resulting in the reduction of intracellular ice crystal formation and cell damage. Similarly, heterologous expression of wild-type (WT) mammalian aquaporin-3 (AQP3) in vertebrate oocytes and embryos enhances both the efflux of water and influx of cryoprotectants into the cells [31,34,35,37-41]. In the case of mouse oocytes, this method ameliorates oocyte tolerance to freezing and survival after thawing [35].

The increased permeability mediated by the artificial expression of AQP3 may be however insufficient for cryosurvival of cells or multicellular systems with large volumes, such as oocytes and embryos from lower vertebrates [39]. This may be related to a decreased translation efficiency of AQP3 when expressed in an heterologous system and/or a limited water and solute permeability of AQP3 [39,42,43]. In addition, AQP3 is pH-dependant [44], and therefore, an effect of pH on AQP3-mediated water and cryoprotectant transport is likely to occur. This additional obstacle should be considered when using AQP3 in cryobiology as the intracellular pH is altered during volumetric changes in cells exposed to hypertonic or hypotonic environments [45-47]. Furthermore, the intracellular pH can be affected by the presence of cryoprotectants within the cell as some of them are dipolar aprotic or protic solvents and act as hydrogen bond acceptors or proton donors, and affect the acid-base equilibrium in mixed solvent systems [46]. Therefore, exploiting further the possibilities of using aquaporins in cryobiology, it would be useful to develop aquaporins with enhanced permeability or pH insensitivity, by investigating GLPs of non-mammalian origin that may be more permeable to water and cryoprotectants than mammalian AQP3.

Recently, we have characterized the genomic repertoire of zebrafish (*Danio rerio*) aquaporins which contains seven GLP isoforms: DrAqp3a and -3b, DrAqp7, DrAqp9a and -9b, and DrAqp10a and -10b [48]. Functional expression in *Xenopus laevis *oocytes demonstrated that all the zebrafish GLPs transport water, glycerol and urea in a similar manner as the mammalian orthologs [48], and therefore these channel proteins are of potential interest for cryobiology. In the present study, we have investigated both the pH sensitivity and cryoprotectant permeability of the zebrafish GLPs by using the *X. laevis *oocyte expression system. Interestingly, we found that both DrAqp3a and -3b isoforms are much more permeable to ethylene glycol than to glycerol or propylene glycol at neutral or alkaline pH, while all other zebrafish GLPs, as well as human AQP3, are more permeable to glycerol. Since ethylene glycol is a suitable cryoprotectant, we further engineered two DrAqp3 mutants that were insensitive to changes in pH or exhibit enhanced ethylene glycol permeability.

## Results

### Effect of pH on water and cryoprotectant permeability of zebrafish GLPs and human AQP3

**Figure 1:**
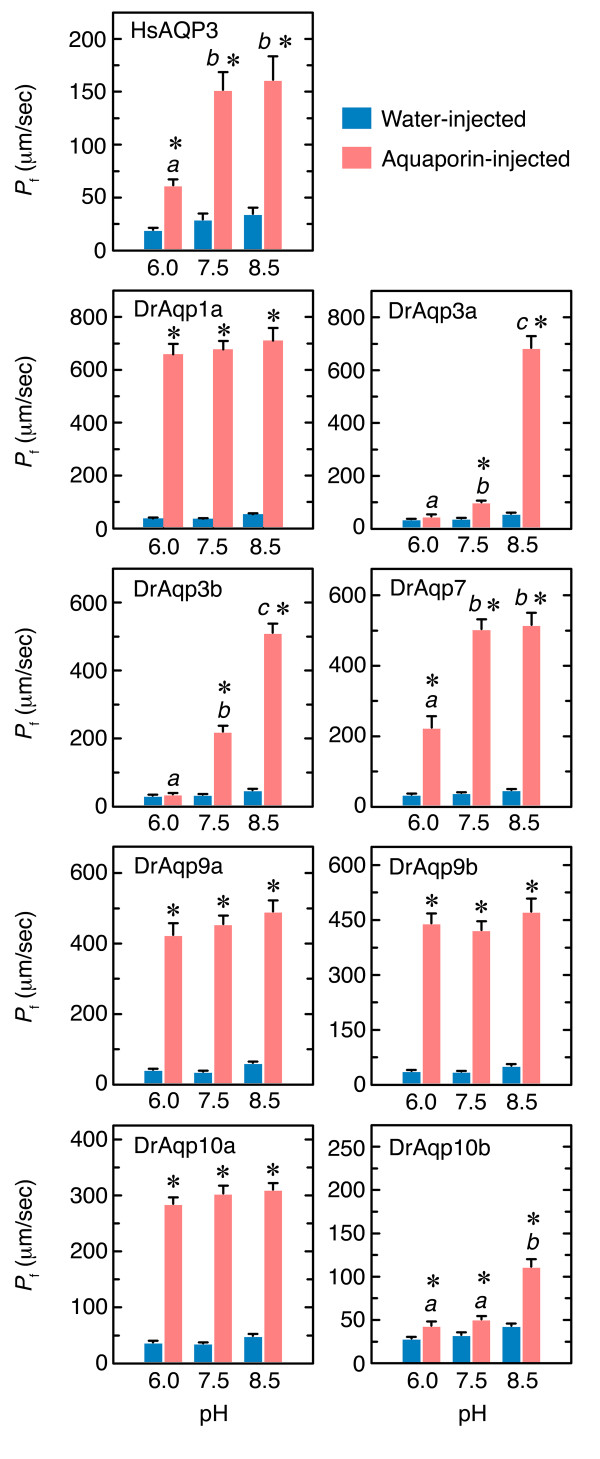
**Effect of pH on zebrafish GLPs and Aqp1a and human AQP3 expressed in *X. laevis *oocytes**. Oocytes were injected with 1 ng (DrAqp3b, -7, -9a, -9b, -10a, -1a or HsAQP3) or 10 ng (DrAqp3a and -10b) cRNA, or with 50 nl of water (controls). The osmotic water permeability (*P*_f_) was measured in 10 times diluted MBS after 15 min of exposure to isotonic MBS at pH 6, 7.5 or 8.5. The swelling experiments were also performed at the corresponding pH. Data are the means ± SEM of three experiments (*n *= 10-12 oocytes for each aquaporin). Bars with different superscript in each panel indicate significant differences (ANOVA, *p *< 0.05) in *P*_f _between aquaporin-injected oocytes. The asterisks denote significant differences with respect the control oocytes at a given pH (Student's *t *test, *p *< 0.05).

The effect of external pH on water permeability of *X. laevis *oocytes injected with capped RNA (cRNA) encoding DrAqp3a -3b, -7, -9a, -9b, -10a, or -10b was determined by measuring the rate of swelling in hypotonic modified Barth's medium (MBS) (Figure 1). For comparison, oocytes were injected with DrAqp1a or human AQP3 (HsAQP3). The pH of the incubation medium affected the osmotic water permeability (*P*_f_) of oocytes expressing HsAQP3, DrAqp3a, -3b, -7, and -10b, whereas the *P*_f _of oocytes injected with DrAqp1a, -9a, -9b, and -10a did not changed significantly within the range of pH tested. However, the effect of pH on oocyte *P*_f _relied on the specific expressed aquaporin. Thus, the *P*_f _of oocytes expressing DrAqp7 was inhibited by approximately 50% at pH 6, whereas the DrAqp10b oocytes, exhibiting very low water permeability at pH 6 and 7.5 (approximately 1.5-fold increase in both cases with respect the control oocytes), showed an enhanced *P*_f _at pH.8.5 (2.5-fold increase). For oocytes expressing HsAQP3 or zebrafish AQP3 isoforms, the *P*_f _was inhibited at acidic pH but in a different manner. The *P*_f _of HsAQP3 oocytes was only partially inhibited (by approximately 55%) at pH 6, whereas it remained similar at neutral and basic pH, as it has been described previously [44]. In contrast, the *P*_f _of DrAqp3a and -3b oocytes was completely abolished at pH 6, and was higher at basic than at neutral pH. Thus, in DrAqp3a oocytes the *P*_f _increased by 5- and 10-fold with respect the control oocytes at pH 7.5 and 8.5, respectively, whereas the *P*_f _of DrAqp3b oocytes increased by 2.5- and 11-fold at pH 7.5 and 8.5, respectively. It thus appears that human AQP3 and the zebrafish orthologs exhibit different sensitivity to pH.

The permeability of *X. laevis *oocytes expressing HsAQP3 or zebrafish GLPs to the cryoprotectants glycerol, propylene glycol and ethylene glycol (*P*_Gly_, *P*_PG _and *P*_EG_, respectively) at different pH was determined volumetrically. In these experiments, control and aquaporin-expressing oocytes were exposed to isotonic solutions containing 160 mM of the respective solutes and changes in oocyte volume were recorded during 1 min. In general, the solute permeability (*P*_S_) of control oocytes and of oocytes expressing the different GLPs was affected by changes in pH (Table 1). Calculation of the oocyte *P*_S _as fold increase with respect that of the controls revealed that the pH changed the *P*_S _in a manner similar to the *P*_f_, except for oocytes expressing HsAQP3 which *P*_EG _remained unchanged regardless of the pH of the medium (Table 1). Oocytes expressing DrAqp7, -9a, -9b, -10a and -10b, or HsAQP3, were significantly (*p *< 0.05) more permeable to glycerol than propylene glycol or ethylene glycol at pH 7.5 (Table 1). In contrast, DrAqp3a or -3b oocytes exhibited significantly (*p *< 0.05) higher permeability to ethylene glycol than to the other cryoprotectants at pH 7.5 or 8.5. At pH 8.5, the *P*_EG _of DrAqp3b oocytes was greatly enhanced resulting in bursting of the oocytes. To prevent this, oocytes were exposed to 100 mM ethylene glycol instead of 160 mM, confirming the stimulatory effect of increasing pH on the *P*_EG _of oocytes expressing DrAqp3b (data not shown).

**Table 1:** Cryoprotectant permeability (*P*_S_) of *X. laevis *oocytes expressing human AQP3 or zebrafish GLPs in isotonic solutions at different pH containing 160 mM of solutes

	*P*_Gly _(x 10^-3 ^cm/min)			*P*_PG _(x 10^-3 ^cm/min)			*P*_EG _(x 10^-3 ^cm/min)		
			
	pH 6.0	pH 7.5	pH 8.5	pH 6.0	pH 7.5	pH 8.5	pH 6.0	pH 7.5	pH 8.5
HsAQP3	9.95 ± 1.17 ^a^ (1.64 ± 0.29)	35.22 ± 3.48 ^c^ (2.07 ± 0.32)	49.62 ± 5.96 ^e^ (3.13 ± 0.38)	10.05 ± 1.64 ^a^ (2.47 ± 0.29)	28.93 ± 3.62 ^c^ (3.13 ± 0.34)	38.14 ± 2.87 ^cd^ (5.23 ± 0.56)	18.34 ± 2.32 ^b^ (1.97 ± 0.31)	32.38 ± 2.87 ^c^ (4.30 ± 0.40)	36.53 ± 3.28 ^c^ (4.89 ± 0.62)
DrAqp3a	2.21 ± 0.40 ^a^ (1.45 ± 0.20)	4.64 ± 0.97^b^ (2.10 ± 0.30)	32.64 ± 4.53 ^d^ (3.33 ± 0.48)	5.73 ± 0.91 ^b^ (2.41 ± 0.21	16.09 ± 1.86 ^c^ (2.64 ± 0.28)	71.08 ± 6.85 ^e^ (5.41 ± 0.46)	3.76 ± 0.69 ^ab^ (1.83 ± 0.29)	20.63 ± 3.18 ^c^ (3.70 ± 0.66)	90.04 ± 6.02 ^f^ (3.77 ± 0.39)
DrAqp3b ^1^	1.71 ± 0.43 ^a^ (1.11 ± 0.25)	12.81 ± 1.24 ^b^ (1.47 ± 0.31)	57.37 ± 3.03 ^d^ (2.93 ± 0.63)	1.92 ± 0.42 ^a^ (1.54 ± 0.33)	28.27 ± 2.51 ^c^ (3.77 ± 0.37)	70.08 ± 4.48 ^e^ (7.84 ± 0.98)	1.46 ± 0.29 ^a^ (1.15 ± 0.71)	79.22 ± 5.70 ^e^ (4.50 ± 0.50)	_
DrAqp7	nd	97.99 ± 7.16 ^c^ (3.85 ± 0.55)	nd	nd	40.76 ± 3.37 ^a^ (3.34 ± 0.35)	nd	Nd	50.55 ± 2.81 ^b^ (4.53 ± 0.57)	nd
DrAqp9a	nd	27.58 ± 1.85 ^a^ (1.76 ± 0.16)	nd	nd	29.40 ± 3.19 ^a^ (5.06 ± 0.37)	nd	Nd	61.62 ± 4.45 ^b^ (7.75 ± 0.60)	nd
DrAqp9b	nd	52.48 ± 3.54 ^b^ (1.92 ± 0.24)	nd	nd	31.27 ± 4.39 ^a^ (3.94 ± 0.38)	nd	Nd	56.03 ± 4.30 ^b^ (9.24 ± 0.70)	nd
DrAqp10a	nd	31.05 ± 3.01 ^b^ (1.60 ± 0.23)	nd	nd	13.02 ± 1.45 ^a^ (4.45 ± 0.41)	nd	Nd	19.14 ± 1.17 ^a^ (6.99 ± 0.61)	nd
DrAqp10b	1.15 ± 0.16 ^a^ (1.29 ± 0.21)	2.29 ± 0.32 ^b^ (1.71 ± 0.26)	17.48 ± 2.25 ^ef^ (2.84 ± 0.52)	2.45 ± 0.50 ^bc^ (2.04 ± 0.30)	3.66 ± 0.51 ^c^ (2.59 ± 0.30)	13.21 ± 1.35 ^e^ (4.59 ± 0.56)	3.30 ± 0.99 ^bc^ (1.93 ± 0.30)	6.70 ± 0.82 ^d^ (3.67 ± 0.74)	18.58 ± 1.26 ^f^ (3.77 ± 0.41)

Data are the means ± SEM (*n *= 10-12 oocytes per treatment) of 3-4 experiments performed on different batches of oocytes. Due to natural variations in oocyte permeability between batches of oocytes, the *P*_S _of water-injected oocytes (controls) is shown in parenthesis for each aquaporin. Oocytes were injected with 1 ng (DrAqp3b, -7, -9a, -9b, -10a, -1a, or HsAQP3) or 10 ng (DrAqp3a and -10b) cRNA, or with 50 nl of water. Data from the same row with different superscript are statistically significant (ANOVA, *p *< 0.05). nd, not determined.

^1 ^No data available for *P*_EG _at pH 8.5 because DrAqp3b-expressing oocytes burst in 160 mM ethylene glycol.

In order to assure that the effect of pH on the *P*_f _or *P*_EG _of DrAqp3b-expressing oocytes was not affected by the dilution of the ion concentrations in the MBS solution, oocytes were equilibrated in isotonic MBS containing sucrose, and a lower concentration of NaCl, prior to the swelling assays (see Methods). These experiments confirmed that the *P*_f _or *P*_EG _at different pH of oocytes expressing DrAqp3b was identical regardless of the ion concentrations of the bathing solutions (Additional file 1), and therefore subsequent *P*_f _assays were carried out in diluted MBS.

**Figure 2:**
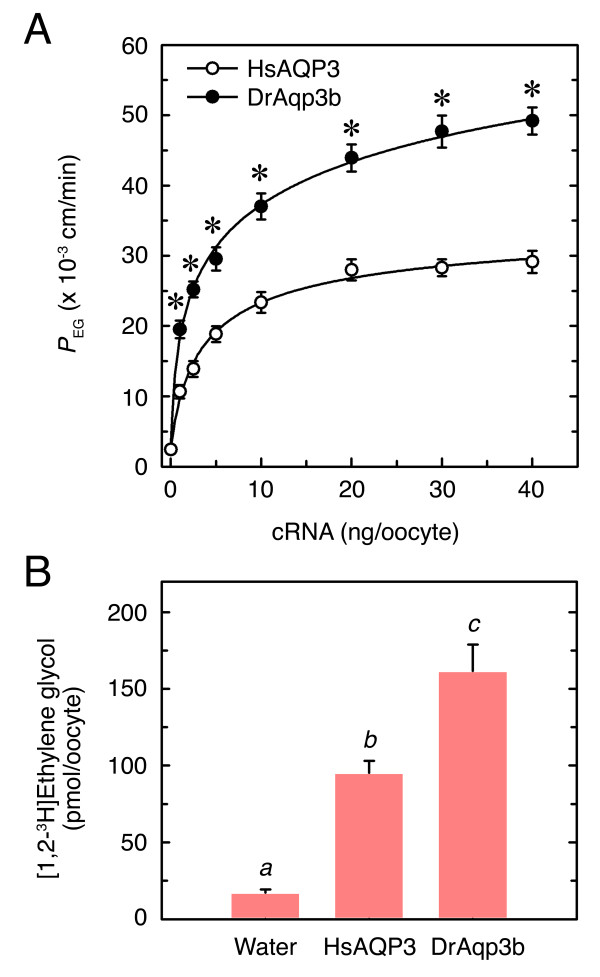
***P*_EG _and ethylene glycol uptake of *X. laevis *oocytes expressing HsAQP3 or DrAqp3b**. (A) *P*_EG _of oocytes expressing different amounts of cRNA (1-40 ng) encoding HsAQP3 or DrAqp3b. Control oocytes were injected with 50 nl of water. The *P*_EG _was measured by swelling measurements during 20 sec in isotonic MBS containing 60 mM of ethylene glycol at pH 7.5. Values are the mean ± SEM of three experiments (*n *= 8-10 oocytes for each aquaporin). Data with an asterisk at the same cRNA dose are significantly different (Student's *t *test, *p *< 0.01). (B) Uptake of radiolabeled ethylene glycol of oocytes injected with 50 nl of water or 5 ng of HsAQP3 or DrAqp3b cRNA. Oocytes were exposed to isotonic MBS containing 1 mM cold ethylene glycol and 5 μM radiolabelled [1,2-^3^H]ethylene glycol for 1 min. Values (mean ± SEM; *n *= 8-10 oocytes) with different superscript are significantly (ANOVA, *p *< 0.01).

To further determine whether the ethylene glycol permeability of oocytes expressing DrAqp3b was higher than that of oocytes expressing HsAQP3, oocytes from the same batch were injected with increasing amounts of cRNA (1 to 40 ng) encoding HsAQP3 or DrAqp3b (Figure 2). Determination of the oocyte *P*_EG _for each cRNA dose at pH 7.5 showed that the *P*_EG _of both HsAQP3- and DrAqp3b-injected oocytes reached a plateau with 20 ng cRNA, but the *P*_EG _of DrAqp3b oocytes was significantly (*p *< 0.01) higher than that of the HsAQP3 oocytes at all tested cRNA doses (Figure 2A). Since the oocyte expression system was apparently saturated with the same cRNA dose in HsAQP3- and DrAqp3b-expressing oocytes, the differences in *P*_EG _were likely not derived from differences in translation efficiency. To confirm that DrAqp3b-expressing oocytes permeated ethylene glycol more efficiently than those expressing HsAQP3, oocytes injected with 20 ng DrAqp3b or HsAQP3 cRNA were exposed to radiolabeled ethylene glycol. These experiments showed that the uptake of isotope-labeled ethylene glycol by DrAqp3b oocytes was approximately 70% higher than in HsAQP3 oocytes (Figure 2B). Altogether, these results suggested that DrAqp3b was more permeable to ethylene glycol than to propylene glycol or glycerol, and that DrAqp3b oocytes permeated ethylene glycol more efficiently than those expressing HsAQP3.

### Development of DrAqp3b mutants pH insensitive or with enhanced water and ethylene glycol permeability

**Figure 3:**
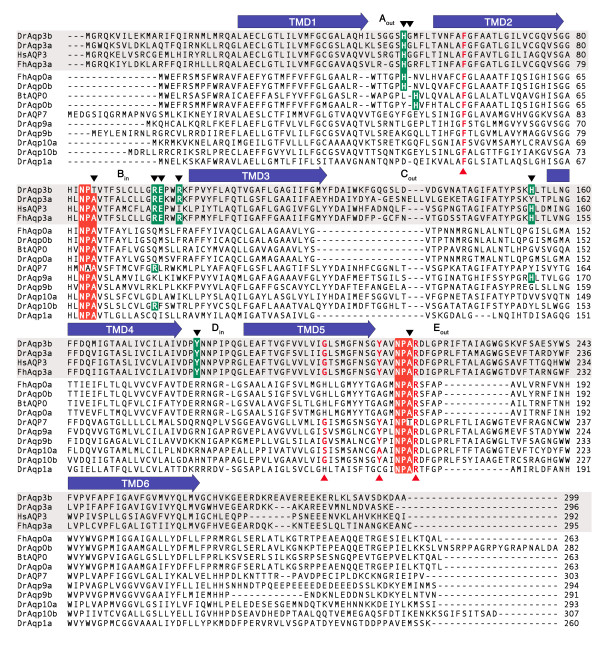
**Amino acid sequence alignment of zebrafish GLPs, Aqp1a and Aqp0a with mammalian and teleost orthologs**. Amino acid sequence alignment of representative GLPs and water-selective aquaporins of teleosts and mammals: *Danio rerio *Aqp0a (DrAqp0a; FJ666326), -0b (FJ655389), -3a (EU341833), -3b (EU341832), -7 (FJ655385), -9a (FJ655387), -9b (EU341835), -10a (FJ655388), -10b (EU341836), and -1a (AY626937), *Fundulus heteroclitus *Aqp0a (FhAqp0; AF191906) and -3a (ACI49539), *Homo sapiens *AQP3 (HsAQP3; BC013566), and *Bos taurus *AQP0 (BtAQP0; NM_173937). Predicted transmembrane domains (TMD1-6) of DrAqp3b are annotated by blue arrows, and external (out) and internal (in) loops are indicated. The two NPA motifs are shaded in red, whereas the four residues forming the aromatic/arginine (ar/R) constriction in zebrafish GLPs (Phe, Gly/Ser, Tyr/Ala, and Arg) [48] are pointed by red arrowheads. Potential residues involved in pH sensitivity in AQP0 and -3 orthologs are shaded in green, and mutated residues in DrAqp3b are indicated by black arrowheads.

**Figure 4:**
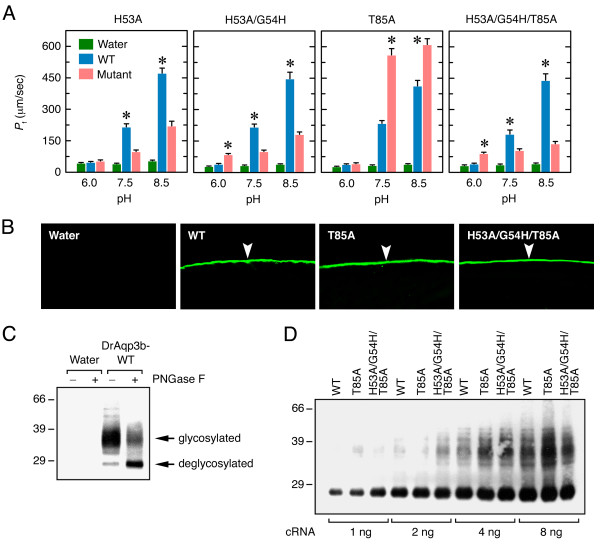
**Functional characterization of DrAqp3b mutants**. (A) *P*_f _of control oocytes (water-injected) and oocytes expressing 1 ng cRNA encoding wild-type DrAqp3b (DrAqp3b-WT) or different DrAqp3b mutants at different pH. Values are the mean ± SEM of three experiments (*n *= 8-10 oocytes per construct). The asterisks denote significant differences between WT and mutant DrAqp3b at a given pH (Student's *t *test, *p *< 0.05). (B) Immunofluorescence microscopy of water-injected oocytes (control), or oocytes expressing DrAqp3b-WT, -H53A, -H53A/G54H, -T85A and -H53A/G54H/T85A using an affinity purified anti-DrAqp3b antiserum. The arrowhead points to the oocyte plasma membrane. (C) Immunoblot of total membrane protein extracts of control oocytes or oocytes expressing DrAqp3b-WT treated or not with N-glycosidase F (PNGase F). The arrows indicate glycosylated and deglycosylated forms of DrAqp3b-WT. (D) Representative immunoblot of plasma membrane protein extracts of oocytes expressing increasing amounts of cRNA (1-8 ng) encoding DrAqp3b-WT, -T85A and -H53A/G54H/T85A treated with PNGaseF.

The finding that both zebrafish Aqp3 isoforms were highly permeable to ethylene glycol prompted us to design Aqp3 mutant constructs that would be pH insensitive or show enhanced ethylene glycol permeability. The design of the mutants was based on the DrAqp3b deduced amino acid sequence (Figure 3 andTable 2) since the corresponding cRNA was expressed apparently more efficiently in *X. laevis *oocytes than that of DrAqp3a. To characterize the DrAqp3b-WT and mutants, their cRNAs were expressed in oocytes and the *P*_f _monitored at different pH (Figure 4A andAdditional file 2).

**Table 2:** Amino acid sequences of zebrafish wild-type (WT) and mutated Aqp3b

Construct	Loop A	Loop B	Loop C	Loop D	Loop E
DrAqp3b-WT	HILSGGS**HG**MFLTV	SGGHINP**T**VTFSLCLLG**RE**PW**R**KFP	FATYPSK**H**LTLL	IVDP**Y**NNPIPQGLEA	AVNP**A**RDLGPRIFTAIAG
H53A	HILSGGS**A**GMFLTV	SGGHINPTVTFSLCLLGREPWRKFP	FATYPSKHLTLL	IVDPYNNPIPQGLEA	AVNPARDLGPRIFTAIAG
G54A	HILSGGSH**A**MFLTV	SGGHINPTVTFSLCLLGREPWRKFP	FATYPSKHLTLL	IVDPYNNPIPQGLEA	AVNPARDLGPRIFTAIAG
H53A/G54H	HILSGGS**AH**MFLTV	SGGHINPTVTFSLCLLGREPWRKFP	FATYPSKHLTLL	IVDPYNNPIPQGLEA	AVNPARDLGPRIFTAIAG
H53A/G54H/T85A	HILSGGS**AH**MFLTV	SGGHINP**A**VTFSLCLLGREPWRKFP	FATYPSKHLTLL	IVDPYNNPIPQGLEA	AVNPARDLGPRIFTAIAG
T85A	HILSGGSHGMFLTV	SGGHINP**A**VTFSLCLLGREPWRKFP	FATYPSKHLTLL	IVDPYNNPIPQGLEA	AVNPARDLGPRIFTAIAG
R95A	HILSGGSHGMFLTV	SGGHINPTVTFSLCLLG**A**EPWRKFP	FATYPSKHLTLL	IVDPYNNPIPQGLEA	AVNPARDLGPRIFTAIAG
E96A	HILSGGSHGMFLTV	SGGHINPTVTFSLCLLGR**A**PWRKFP	FATYPSKHLTLL	IVDPYNNPIPQGLEA	AVNPARDLGPRIFTAIAG
R99A	HILSGGSHGMFLTV	SGGHINPTVTFSLCLLGREPW**A**KFP	FATYPSKHLTLL	IVDPYNNPIPQGLEA	AVNPARDLGPRIFTAIAG
H154A	HILSGGSHGMFLTV	SGGHINPTVTFSLCLLGREPWRKFP	FATYPSK**A**LTLL	IVDPYNNPIPQGLEA	AVNPARDLGPRIFTAIAG
G54H/H154A	HILSGGSH**H**MFLTV	SGGHINPTVTFSLCLLGREPWRKFP	FATYPSK**A**LTLL	IVDPYNNPIPQGLEA	AVNPARDLGPRIFTAIAG
H53A/H154A	HILSGGS**A**GMFLTV	SGGHINPTVTFSLCLLGREPWRKFP	FATYPSK**A**LTLL	IVDPYNNPIPQGLEA	AVNPARDLGPRIFTAIAG
H53A/G54H/H154A	HILSGGS**AH**MFLTV	SGGHINPTVTFSLCLLGREPWRKFP	FATYPSK**A**LTLL	IVDPYNNPIPQGLEA	AVNPARDLGPRIFTAIAG
A217T	HILSGGSHGMFLTV	SGGHINPTVTFSLCLLGREPWRKFP	FATYPSKHLTLL	IVDPYNNPIPQGLEA	AVNP**T**RDLGPRIFTAIAG
T85A/A217T	HILSGGSHGMFLTV	SGGHINP**A**VTFSLCLLGREPWRKFP	FATYPSKHLTLL	IVDPYNNPIPQGLEA	AVNP**T**RDLGPRIFTAIAG
Y182A	HILSGGSHGMFLTV	SGGHINPTVTFSLCLLGREPWRKFP	FATYPSKHLTLL	IVDP**A**NNPIPQGLEA	AVNPARDLGPRIFTAIAG

Amino acid sequence of the five loops of WT and DrAqp3b mutants. The residues mutated and the substitutions are underlined in bold.

To design DrAqp3b constructs encoding aquaporins potentially pH insensitive, we targeted conserved residues that have been suggested to contribute to pH sensitivity in vertebrate and plant aquaporins, such as His, Arg, Glu and Tyr [49-53] (Table 2 and Figure 3). Within the external loop A, fish and human AQP3 contain a His residue (His^53 ^in DrAqp3b) that is conserved in killifish (*Fundulus heteroclitus*) Aqp0a (His^39^) (Figure 3), which is, as AQP3, less permeable at acidic pH [51]. However, bovine (*Bos taurus*) AQP0, where the His in loop A is shifted one position (His^40^) as compared to FhAqp0a (Figure 3), is more permeable at acidic pH [50]. Expression of DrAqp3b-H53A (Figure 4A) or -G54H (Additional file 2) in oocytes led to a global decrease in water permeability with respect DrAqp3b-WT oocytes and did not affect pH sensitivity. By moving the His from position 53 to 54 (DrAqp3b-H53A/G54H), oocyte permeability at pH 6 was increased by 97% (*p *< 0.05) with respect to DrAqp3b-WT oocytes. However, the permeability at pH 7.5 and 8.5 of oocytes expressing this mutant was reduced when compared with DrAqp3b-WT oocytes (Figure 4A).

In the external loop C, the His^154 ^of DrAqp3b is conserved in FhAqp3a and HsAQP3 (Figure 3). This residue in HsAQP3 has been proposed to participate in the pH regulation of water permeability [49]. In DrAqp3b, His^154 ^was necessary for water permeability, since the *P*_f _at pH 6 and 7.5 of oocytes expressing DrAqp3b-H154A, -G54H/H154A, -H53A/H154A or -H53A/G54A/H154A, was strongly diminished with respect to that of the DrAqp3-WT oocytes, although it was slightly less inhibited at pH 8.5 (Additional file 2).

Since Tyr residues have been reported to be implied in the pH sensitivity of HsAQP3 [49], and Arg residues may create electrostatic barriers that regulate aquaporin permeability [52], we also investigated the effect of mutations in these residues. The Tyr^182 ^in the internal loop D of DrAqp3b is conserved in both fish and human AQP3 but not in other zebrafish GLPs and DrAqp1a (Figure 3). In addition, several Arg and Glu residues in the internal loop B (Arg^95^, Glu^96 ^and Arg^99 ^in DrAqp3b) are highly conserved in vertebrate AQP3, of which Arg^95 ^is retained in DrAqp7 and -10b that show decreased water permeability at pH 6 (Figure 1). However, all the mutations performed at these sites (DrAqp3b-R95A, -E96A, -R99A, and -Y182A) did not affect significantly the *P*_f _at any pH (Additional file 2), suggesting that these residues are not essential for water permeability or pH sensitivity in DrAqp3b.

The NPA motifs, together with the aromatic residue/arginine (ar/R) constriction region (Figure 3), are believed to be involved in proton exclusion and channel selectivity of aquaporins [54,55]. Since DrAqp3b exhibits a particular feature within the first NPA box, apparently conserved in the Aqp3b of other teleosts [48], where the Ala residue is replaced by a Thr (Figure 3), we investigated whether the Thr^85 ^of DrAqp3b can affect its permeability. The first NPT motif of DrAqp3b was mutated into NPA (DrAqp3b-T85A; Table 2) and expressed in oocytes. Two other mutants were produced bearing two NPT motifs (DrAqp3b-A217T) or NPA-NPT motifs (DrAqp3b-T85A/A217T). The oocytes expressing DrAqp3b-T85A showed a significant (*p *< 0.05) 2.4- and 1.5-fold increase in *P*_f _with respect oocytes expressing DrAqp3b-WT at pH 7.5 and 8.5, respectively, and had similar *P*_f _at neutral and alkaline pH (Figure 4A). Thus, substitution of Thr by Ala in the first NPA box of DrAqp3b increased water permeability and completely abolished the stimulatory effect of alkaline pH, although permeability was still inhibited at pH 6. Expression of the DrAqp3b-A217T and -T85A/A217T mutants in oocytes decreased the *P*_f _with respect the oocytes expressing DrAqp3b-WT and did not affect the pH sensitivity of DrAqp3b (Additional file 2).

Since the DrAqp3b-H53A/G54H mutant displayed higher water permeability at pH 6 than the WT, and the DrAqp3b-T85A was more permeable and not affected by alkaline pH, we finally designed and characterized a triple mutant DrAqp3b-H53A/G54H/T85A (Table 2). Interestingly, the *P*_f _of oocytes expressing this construct was insensitive to pH, being 2.2-fold higher than that of the DrAqp3b-WT oocytes at pH 6 (Figure 4A). The *P*_f _of these oocytes was, however, 2- and 3.3-times lower, respectively, than that of DrAqp3b-WT oocytes at pH 7.5 and 8.5.

The mutagenesis experiments rendered two constructs, DrAqp3b-T85A and - H53A/G54H/T85A, that encoded aquaporins with enhanced water permeability at pH 7.5 and 8.5, or insensitive to pH, respectively. In order to characterize the expression levels and subcellular localization of the protein products of these mutants in *X. laevis *oocytes, immunocytochemistry and Western blotting were carried out using an affinity-purified antibody against DrAqp3b. Immunofluorescence microscopy of oocytes expressing DrAqp3b-WT, -T85A or -H53A/G54H/T85A revealed that in all cases the corresponding proteins were localized exclusively in the plasma membrane (Figure 4B). Western blot analysis of total membrane extracts of DrAqp3b-WT injected oocytes showed a broad and intense immunoreactive band of approximately 33-40 kDa (Figure 4C). This band corresponded to a glycosylated form of DrAqp3b, since treatment with N-Glycosidase F (PNGase F) rendered a product of approximately 29 kDa. Immunoblotting of plasma membrane extracts treated with PNGase F of oocytes injected with increasing amounts of DrAqp3b-WT, -T85A or -H53A/G54H/T85A showed that similar amounts of the protein products were accumulated at the oocyte plasma membrane (Figure 4D). However, the DrAqp3b-H53A/G54H/T85A construct resulted in sligthly more protein at the plasma membrane in comparison with the other constructs when 1 ng cRNA was injected into the oocytes (Figure 4D). Since oocytes expressing this mutant had lower *P*_f _than the DrAqp3b-WT and -T85A oocytes at pH 7.5, our observations confirmed that the differences in *P*_f _between oocytes expressing DrAqp3b-WT or the mutants were not derived from differences in translation or in the accumulation of the protein products in the oocyte membranes.

**Figure 5:**
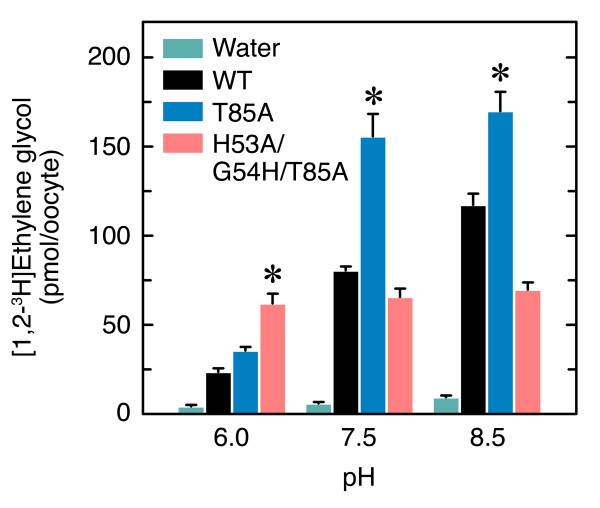
**Ethylene glycol uptake of DrAqp3b-WT and mutants at different pH**. Uptake of radiolabeled ethylene glycol by oocytes injected with water or with 5 ng of DrAqp3b-WT, -T85A or -H53A/G54H/T85A cRNA was determined as in Figure 2. Data (mean ± SEM; *n *= 8-10 oocytes) with an asterisk are significantly (ANOVA, *p *< 0.01) different from the DrAqp3b-WT.

The permeability of oocytes expressing DrAqp3b-WT, -T85A or -H53A/G54H/T85A to ethylene glycol was finally investigated by radioactive uptake assays as described previously (Figure 5). The data indicated than the permeability to ethylene glycol of oocytes expressing the different constructs followed the same pattern as the *P*_f_. Thus, the DrAqp3b-H53A/G54H/T85A oocytes showed higher ethylene glycol uptake than the other oocytes at pH 6, and the uptake did not change at higher pH. In contrast, at pH 7.5 and 8.5 the DrAqp3b-T85A oocytes showed the highest ethylene glycol uptake.

### Permeability of DrAqp3b mutants under hypertonic conditions

**Figure 6:**
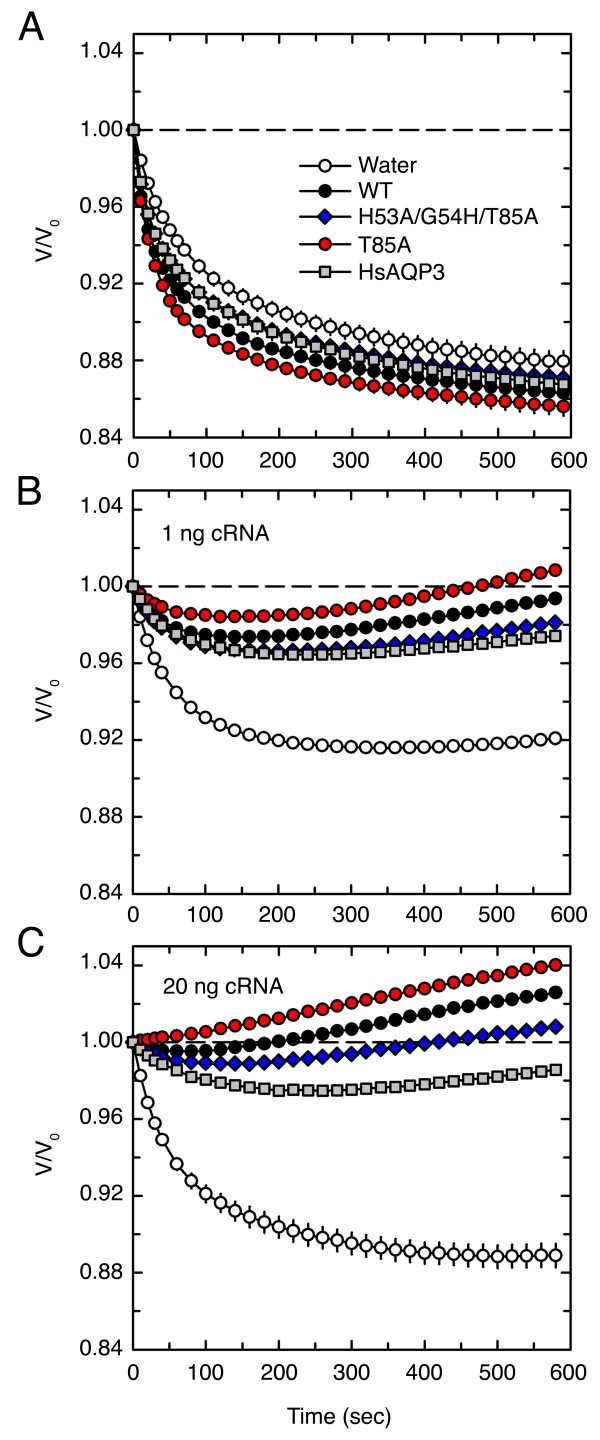
**Changes in cell volume of oocytes expressing DrAqp3b-WT, DrAqp3b mutants or HsAQP3 in hypertonic solutions**. (A) Oocytes expressing 1 ng of DrAqp3b-WT, -T85A, -H53A/G54H/T85A or HsAQP3 were exposed to 0.9 M sucrose in MBS for 10 min. (B-C) Oocytes expressing 1 (B) or 20 (C) ng of the same constructs were exposed to 1.3 M ethylene glycol in MBS for 10 min. Data from all panels are means ± SEM of 15-24 oocytes from 3-4 different batches.

The permeability to water and ethylene glycol of oocytes expressing HsAQP3, DrAqp3b-WT, -T85A or -H53A/G54H/T85A was subsequently determined in hypertonic solutions (Figure 6). We first investigated the hydraulic conductivity (*L*_p_) of oocytes expressing 1 ng of cRNA of the different constructs immersed in a sucrose solution (0.9 M). Oocytes from all groups shrank quickly during approximately the first 2 min of sucrose exposure, and the shrinkage decreased thereafter, indicating that they were slowly reaching the equilibration state (Figure 6A). As expected, aquaporin-expressing oocytes shrank faster than the controls, although the oocytes expressing DrAqp3b-T85A shrank more rapidly than the oocytes expressing the other constructs (Figure 6A). Since we observed that the shrinkage of oocytes in 0.9 M sucrose was linear only during the first 2 min, we calculated the *L*_p _after 2 and 10 min; the *L*_p _at 2 min being more comparable to the *P*_f _previously determined under hypotonic conditions. Accordingly, the *L*_p _values after 2 min of the DrAqp3b-T85A oocytes were the highest, followed by the oocytes expressing DrAqp3b-WT, -H53A/G54H/T85A, HsAQP3 and water-injected oocytes (Table 3). After 10 min, the *L*_p _of the DrAqp3b-T85A oocytes remained higher than that of the other groups, whereas the *L*_p _of the DrAqp3b-WT, -H53A/G54H/T85A and HsAQP3 oocytes was similar and higher than the control oocytes (Table 3). Therefore, the results confirmed that the DrAqp3b-T85A mutant was also the most permeable to water under hypertonic conditions.

**Table 3:** Hydraulic conductivity (*L*_p_) of *X. laevis *oocytes expressing wild-type (WT) DrAqp3b, DrAqp3b mutants or HsAQP3 determined after 2 or 10 min in a sucrose solution

	*L*_p _(μm/min/atm)	
	
	2 min	10 min
Water	1.40 ± 0.06 ^a^	0.69 ± 0.03 ^a^
DrAqp3b-WT	2.15 ± 0.06 ^c^	0.89 ± 004 ^b^
DrAqp3b-H53A/G54H/T85A	1.94 ± 0.08 ^b^	0.88 ± 004 ^b^
DrAqp3b-T85A	2.57 ± 0.07 ^d^	1.02 ± 0.04 ^c^
HsAQP3	1.93 ± 0.07 ^b^	0.87 ± 0.03 ^b^

Values (mean ± SEM) for water- and aquaporin-injected (1 ng cRNA) oocytes are calculated from the data in Figure 6A. Data from the same column with different superscript are statistically significant (ANOVA, *p *< 0.05).

Next, we evaluated the *L*_p _and *P*_EG _of oocytes expressing the different constructs under a high concentration gradient of ethylene glycol (1.3 M) for 10 min. For these experiments, we used oocytes expressing 1 or 20 ng of cRNA. The relative volume changes of the oocytes under these conditions revealed that water-injected oocytes shrank and did not swell for almost 10 min, suggesting that ethylene glycol permeated oocytes quite slowly (Figure 6B and 6C). HsAQP3- and DrAqp3b-H53A/G54H/T85A-expressing oocytes (1 ng cRNA) shrank slightly less than controls, and then swelled slowly, indicating a higher permeability to the cryoprotectant (Figure 6B). The DrAqp3b-WT oocytes shrank less and swelled more than those injected with DrAqp3b-H53A/G54H/T85A- or HsAQP3, whereas oocytes expressing DrAqp3b-T85A exhibited few shrinkage and the fastest re-swelling (Figure 6B). Accordingly, the *L*_p _values of the DrAqp3b-T85A oocytes in 1.3 M ethylene glycol were the highest, followed by the DrAqp3b-WT, -H53A/G54H/T85A, HsAQP3, and control oocytes (Table 4). This was in agreement with the data obtained using oocytes exposed to 0.9 M sucrose. The *P*_EG _values also corroborated previous volumetric and ethylene glycol uptake experiments, since the *P*_EG _of DrAqp3b-T85A oocytes was significantly (*p *< 0.05) higher than that of the oocytes expressing the other constructs or injected with water (Table 4). The reflection coefficients (σ), calculated using the Kedem-Katchalsky model (see Methods), were the lowest in aquaporin-expressing oocytes (data not shown), suggesting that water and solute permeate through the same pore.

**Table 4:** Hydraulic conductivity (*L*_p_) and ethylene glycol permeability (*P*_EG_) of *X. laevis *oocytes expressing wild-type (WT) DrAqp3b, two DrAqp3b mutants or HsAQP3, in hypertonic ethylene glycol solution

	*L*_p _(μm/min/atm)	*P*_EG _(x 10^-3 ^cm/min)
Water	0.36 ± 0.02 ^a^	0.47 ± 0.03 ^a^
DrAqp3b-WT (1 ng)	2.15 ± 0.14 ^c^	17.59 ± 0.83 ^d^
DrAqp3b-H53A/G54H/T85A (1 ng)	1.55 ± 0.13 ^b^	13.06 ± 0.72 ^c^
DrAqp3b-T85A (1 ng)	2.64 ± 0.15 ^d^	25.49 ± 1.56 ^e^
HsAQP3 (1 ng)	1.31 ± 0.08 ^b^	10.73 ± 0.59 ^b^
DrAqp3b-WT (20 ng)	2.63 ± 0.07 ^d^	40.06 ± 1.22 ^c^
DrAqp3b-H53A/G54H/T85A (20 ng)	2.27 ± 0.07 ^c^	36.58 ± 1.74 ^c^
DrAqp3b-T85A (20 ng)	3.24 ± 0.08 ^e^	53.91 ± 2.55 ^d^
HsAQP3 (20 ng)	1.96 ± 0.08 ^b^	22.24 ± 0.95 ^b^

Values (mean ± SEM) for water- and aquaporin-injected (1 or 20 ng cRNA) oocytes are calculated from the data in Figure 6B and C. For each cRNA dose, data with different superscript are statistically significant (ANOVA, *p *< 0.05).

In general, oocytes injected with 20 ng of cRNA of each construct shrank less and swelled more rapidly over the 10 min period when compared with oocytes expressing 1 ng (Figure 6C). For the DrAqp3b-T85A oocytes, however, no shrinkage was observed but the oocytes constantly swelled, probably reflecting a very fast permeation of ethylene glycol into the oocyte. Consistently, 20 ng cRNA-injected oocytes had higher *L*_p _and *P*_EG _values than those injected with 1 ng and, as observed before, oocytes expressing DrAqp3b-T85A showed the highest *L*_p _and *P*_EG _values (Table 4).

The internal concentration of ethylene glycol in water-injected oocytes and oocytes expressing the different constructs after immersion in hypertonic ethylene glycol was finally assessed. For these experiments, oocytes were exposed for 10 min to a solution of 1.3 M ethylene glycol at pH 7.5 containing radiolabeled ethylene glycol. The results confirmed that the uptake of the cryoprotectant was highly increased under a concentration gradient (Table 5), since oocytes expressing 1 ng cRNA of the different constructs showed about 150-fold higher uptake rate of ethylene glycol than oocytes expressing 5 ng (Figure 5) or 20 ng (Figure 2) cRNA that were exposed to an isotonic ethylene glycol solution at the same pH. After 10 min in 1.3 M ethylene glycol, the internal concentration of the cryoprotectant in oocytes injected with 1 o 20 ng cRNA of DrAqp3b-T85A was 2.7- and 3.6-times higher, respectively, than that of control oocytes (Table 5). The ethylene glycol concentration in oocytes expressing 1 or 20 ng cRNA of DrAqp3b-WT, -H53A/G54H/T85A, and HsAQP3 was significantly (*p *< 0.05) lower than that of DrAqp3b-T85A oocytes, but still higher than in control oocytes (Table 5).

**Table 5:** Ethylene glycol content (mol/l) of *X. laevis *oocytes expressing different amounts of cRNA encoding wild-type (WT) DrAqp3b, two DrAqp3b mutants, or HsAQP3, after immersion in hypertonic ethylene glycol solution

	cRNA injected	
	
	1 ng	20 ng
Water	0.11 ± 0.004 ^a^ (6050 ± 220)	
DrAqp3b-WT	0.24 ± 0.01 ^c^ (14568 ± 607)	0.34 ± 0.02 ^d^ (21284 ± 1250)
DrAqp3b-H53A/G54H/T85A	0.20 ± 0.01 ^b^ (11980 ± 599)	0.29 ± 0.01 ^c^ (17835 ± 615)
DrAqp3b-T85A	0.33 ± 0.02 ^d^ (20328 ± 1232)	0.43 ± 0.01 ^e^ (27305 ± 635)
HsAQP3	0.20 ± 0.01 ^b^ (11900 ±595)	0.24 ± 0.01 ^b^ (14448 ± 602)

Data are the mean ± SEM (*n *= 12-18 oocytes) of two different experiments. Data in parenthesis are in pmol/oocyte/min. Values from the same column with different superscript are statistically significant (ANOVA, *p *< 0.05).

## Discussion

In the present study, we have characterized the permeability of the complete repertoire of zebrafish GLPs to glycerol, propylene glycol and ethylene glycol in *X. laevis *oocytes. We found that the two zebrafish Aqp3 isoforms, although pH dependent, are selectively more permeable to ethylene glycol than to glycerol or propylene glycol under isotonic conditions. Using this property, apparently unique among vertebrate aquaporins, we have developed two mutant constructs based on the amino acid sequence of DrAqp3b. One of these mutants was pH insensitive when expressed in oocytes, whereas the other showed higher water and ethylene glycol permeability than the WT in both isotonic and hypertonic solutions.

It is known that some mammalian aquaporins are regulated by pH, namely AQP0, -3 and -6 [44,50,56,57]. However, the effect of pH on the permeability of teleost GLPs has not been reported, except for Aqp3a and -3b [58,59]. The results from the present study show that water, glycerol and propylene glycol permeability of oocytes expressing HsAQP3 at pH 6 was reduced to a similar extent as in previous studies, whereas permeability remained unchanged between pH 7.5 and 8.5 [44,49,60]. Zebrafish Aqp3a, -3b, -7 and -10b were affected to a variable degree by changes in pH when expressed in oocytes, although DrAqp3a and -3b were the most affected aquaporins. Thus, water and cryoprotectant permeability of oocytes expressing DrAqp3a or -3b was completely abolished at pH 6 and was highest at pH 8.5. Such an effect of pH on aquaporin permeability, not observed in HsAQP3, has been previously reported for the Aqp3a and -3b from killifish and European eel, respectively [58,59], but its physiological significance remains to be elucidated. The *P*_f _of oocytes expressing DrAqp7 or -10b was also diminished at acidic pH, but only by approximately 50%, a feature that has not been described for the mammalian orthologs.

Determination of the *P*_Gly_, *P*_PG _and *P*_EG _of oocytes expressing the different zebrafish GLPs under isotonic conditions indicated that DrAqp3a and -3b were the only aquaporins more permeable to ethylene glycol than to glycerol or propylene glycol. HsAQP3 was more permeable to glycerol than to the other cryoprotectants under isotonic conditions at pH 7.5 and 8.5, but under high concentration gradients HsAQP3 might transport propylene glycol and ethylene glycol as efficiently as glycerol, as it occurs for rat AQP3 expressed in *X. laevis *oocytes [31]. Therefore, it is possible that in hypertonic solutions oocytes expressing DrAqp3a or -3b may be equally permeable to glycerol, propylene glycol and ethylene glycol. Nevertheless, our results indicate that oocytes expressing DrAqp3b and exposed to isotonic or hypertonic ethylene glycol solutions permeate this cryoprotectant more efficiently than those expressing HsAQP3. The DrAqp3b amino sequence shows the residues forming the ar/R narrowest constriction region conserved in GLPs, but interestingly, it shows an unusual first NPA motif, where Ala is substituted by Thr. This amino acid change may be involved in the increased permeability of DrAqp3b to ethylene glycol with respect glycerol or propylene glycol, since NPA motifs form one of the constriction sites of aquaporins responsible for proton exclusion and size selectivity [61]. However, the observation that changing the NPT motif of DrAqp3b into NPA increased both water and ethylene glycol permeability does not support this supposition. The molecular basis of DrAqp3b ethylene glycol selectivity remains thus unclear and needs to be investigated.

Ethylene glycol is one of the most suitable cryoprotectants in terms of efficiency/toxicity ratio for the preservation of oocytes and embryos from higher vertebrates [13-18], as well as from some fish species [3], and therefore DrAqp3b may be more effective than mammalian AQP3 at increasing the internal concentration of ethylene glycol. Water and solute transport through DrAqp3b is however pH sensitive being maximum at pH 8.5. To counteract this obstacle, we investigated the role of sequence motifs in DrAqp3b potentially critical for pH regulation of water permeability, or that can enhance ethylene glycol permeability. Mutagenesis experiments identified one His residue in loop A of DrAqp3b (His^53^), spanning the outer region of the pore, which precise position within the loop regulated the inhibitory effect of acidic pH on water permeability. These observations are similar to those reported by Németh-Cahalan et al. [51] for the fish and bovine AQP0. In this study, it was suggested that pH can modulate the orientation of water molecules in the pore by titrating external His, which would in turn alter the orientation-dependant electrostatic interactions between water molecules, thus affecting their effective binding energies within the pore. In the present work, we also found that changing the first NPT motif of DrAqp3b into NPA (DrAqp3b-T85A) shifted the maximal permeability to pH 7.5, allowing for higher water and ethylene glycol permeability at pH 7.5 and 8.5 with respect the WT. These findings suggest that Thr^85 ^of DrAqp3b is involved in the sensitivity of this aquaporin to alkaline pH. By combining these two observations, a triple mutant was designed (DrAqp3b-H53A/G54H/T85A) which was no longer pH sensitive (e.g., exhibited similar permeability at all pHs), although it was less permeable at pH 7.5 and 8.5 than the WT.

Increasing cell permeability to water and cryoprotectants, under high concentration gradients, is important for developing and improving cryopreservation protocols [31]. During slow and fast (vitrification) cooling procedures employed for cryopreservation, cells have to be exposed to variable concentrations (around 1.5 M, or >5 M, respectively) of cryoprotectants [14,62]. Our results suggest that mutated DrAqp3b, with improved cryoprotectant permeability, may be useful in cryobiology. The *L*_p _and *P*_EG _of oocytes expressing HsAQP3, DrAqp3b-WT, -T85A- or -H53A/G54H/T85A were measured here in a solution containing 1.3 M ethylene glycol equivalent to that used in slow cooling protocols. For both 1- or 20 ng cRNA-injected oocytes, the *L*_p _and *P*_EG _values were the highest for oocytes expressing DrAqp3b-T85A in agreement with previous observations using isotonic conditions and a solution of 0.9 M sucrose. The *P*_EG _values of oocytes injected with 20 ng of DrAqp3b-WT or -T85A and exposed to 1.3 M ethylene glycol were 40.06 × 10^-3 ^and 53.91 × 10^-3 ^cm/min, respectively, whereas those of the HsAQP3-expressing oocytes were 22.24 × 10^-3 ^cm/min. Yamaji et al. [31] reported a *P*_EG _of 33.5 x10^-3 ^cm/min of *X. laevis *oocytes expressing 40 ng of rat AQP3 and exposed to the same ethylene glycol concentration. Our results therefore suggest that DrAqp3b-WT and -T85A may transport ethylene glycol more efficiently than human or rat AQP3. However, the *L*_p _(0.02 μm/min/atm) and *P*_EG _(0.11 × 10^-3 ^cm/min) of water-injected oocytes reported by Yamaji et al. [31] were 18- and 4-times lower, respectively, than those measured in our study (0.36 μm/min/atm and 0.47 × 10^-3 ^cm/min, respectively) under the same conditions. The causes of these differences remain unknown, but they may be related with the observation that water-injected oocytes shrank faster in our study than those of Yamaji et al. [31] at a similar ethylene glycol concentration. Also, we consistently measured a basal ethylene glycol permeation, under either isotonic or hypertonic solutions, in water-injected oocytes, in both swelling and radiolabeled solute uptake assays.

## Conclusions

In the present study, we have shown that the pH sensitiveness and ethylene glycol permeability of DrAqp3b can be altered by single or triple point mutations in its amino acid sequence. Using this approach, we engineered and functionally characterized two mutant constructs, DrAqp3b-T85A- and -H53A/G54H/T85A, which were either more permeable to water and ethylene glycol than the DrAqp3b-WT or were pH insensitive, respectively. These artificial aquaporins may be useful for cryopreservation of large cells or multicellular systems, and investigations in this direction are underway.

## Methods

### Aquaporin cDNAs and expression constructs

The GenBank accession numbers of the zebrafish aquaporin cDNAs employed in this study were as follows: Aqp1a (AY626937), Aqp3a (EU341833), Aqp3b (EU341832), Aqp7 (FJ655385), Aqp9a (FJ655387), Aqp9b (EU341835), Aqp10a (FJ655388), and Aqp10b (EU341836). The human AQP3 cDNA (GenBank accession number BC013566) was generously provided by Prof. Peter Deen (Radboud University Nijmegen Medical Centre, The Netherlands).

Aquaporin constructs for heterologous expression in *X. laevis *oocytes were generated by subcloning full-length aquaporin cDNAs into the pT7Ts expression vector [63]. Since this vector contains unique *Bgl*II, *Eco*RV and *Spe*I cloning sites to allow the gene of interest to be flanked by the 5' and 3' untranslated regions of the *X. laevis *β-globin gene, compatible *Bgl*II, *Eco*RV or *Spe*I sites were introduced for each aquaporin (depending on the restriction sites identified in the sequence) by PCR using high fidelity polymerase (Easy A, Stratagene). Mutations into the DrAqp3b amino acid sequence were performed on pT7Ts-DrAqp3b plasmid using the Quickchange site-directed mutagenesis kit (Stratagene) and the oligonucleotide primers listed in Additional file 3. Selected clones were sequenced by BigDye Terminator v3.1 cycle sequencing on ABI PRISM 377 DNA analyzer (Applied Biosystems) to confirm that only the desired mutations were produced.

### Sequence analysis

Vertebrate and teleost aquaporin sequences were retrieved from the NCBI database [64]. Amino acid sequence alignments were performed using the ClustalW multiple sequence alignment program [65] employing the full-length amino acid sequence, and were manually optimized using the Bioedit software [66].

### Functional expression in *X. laevis *oocytes

The cRNAs for microinjection were synthesized with T7 RNA polymerase (Roche) from *Xba*I *or Sal*I-linearized pT7Ts-aquaporin (depending on the restriction sites identified in the aquaporin sequence). Isolation of stages V and VI oocytes and microinjection was performed as previously described [63]. Oocytes were transferred to MBS (88 mM NaCl, 1 mM KCl, 2.4 mM NaHCO_3_, 0.82 mM MgSO_4_, 0.33 mM Ca(NO_3_)_2_, 0.41 mM CaCl_2_, 10 mM HEPES, and 25 μg/ml gentamycin, pH 7.5) and injected with 50 nl of distilled water (negative control) or 50 nl of water solution containing 1 to 20 ng of cRNA. One day after injection, oocytes were manually defolliculated and subsequently maintained in MBS at 18°C.

### Determination of oocyte *P*_f_, *P*_GLY_, *P*_PG _and *P*_EG _at different pH

Water and solute permeability of oocytes expressing the different constructs was tested in hypotonic or isotonic MBS (200 mOsm), respectively, at different pH and at room temperature (20°C). For the calculation of *P*_f_, two days after injection the oocytes were transferred to isotonic MBS (200 mOsm) at pH 6, 7.5 or 8.5 for 15 min, and then transferred to 10-fold diluted MBS (20 mOsmol) at the same experimental pH. Oocyte swelling was followed by video microscopy using serial images at 2 sec intervals during the first 20 sec period using a Nikon Color view video camera coupled to a stereomicroscope (SMZ1000, Nikon). The *P*_f _values were calculated taking into account the time-course changes in relative oocyte volume [d(V/V_0_)/dt], the partial molar volume of water (V_W _= 18 cm^3^/mol) and the oocyte surface area (S) using the formula V_0_[d(V/V_0_)/dt]/[SV_W_(Osm_in _- Osm_out_)]. The surface area of the oocyte was considered to be nine times the apparent area because membrane folding [67].

Cryoprotectant (glycerol, propylene glycol and ethylene glycol; Sigma) permeability of water-injected and aquaporin-expressing oocytes was determined also volumetrically in isotonic MBS, where NaCl was replaced by 160 mM of the solutes, at different pH. The osmolarity of the solutions was measured for each experiment with a vapor pressure osmometer (Vapro^® ^5520, Wescor), and adjusted to 200 mOsm with NaCl if necessary. Oocytes were exposed to different pH as indicated above, and oocyte swelling was measured by video microscopy, in this case using serial images at 5 sec intervals during 1 min. In some experiments, the *P*_EG _was estimated using MBS containing 60 or 100 mM ethylene glycol and using serial images of oocyte swelling as for the *P*_f_. The *P*_GLY_, *P*_PG _and *P*_EG _were calculated from oocyte swelling using the formula [d(V/V_0_)dt]/(S/V_0_) [68]. The *P*_s _of oocytes for each of the three cryoprotectants was measured using the same batch of oocytes.

To ensure that the *P*_f _was not affected by ion dilution during oocyte volume measurements [69], the NaCl concentration of MBS was reduced to 78 mM, 50 mM or 25 mM, and sucrose was added to a concentration of 20 mM, 56 mM and 126 mM, respectively, to obtain isotonic solutions as determined with the vapor pressure osmometer. Oocytes were then equilibrated in these solutions at pH 7.5 for 45 min, and subsequently transferred to the same solutions at pH 6, 7.5 or 8.5 for 15 min. Oocyte swelling was measured in these solutions that were made hyposmotic by removing sucrose, and thus the ion concentrations were constant. The same control experiments were carried out for the determination of *P*_EG _at different pH. For this, oocytes were equilibrated in isotonic MBS containing 38 mM NaCl and 100 mM sucrose, which was substituted by 100 mM ethylene glycol for the swelling measurements.

### Determination of the osmotically inactive fraction of *X. laevis *oocytes

The water loss of non-injected *X. laevis *oocytes was assessed by two types of experiments performed at pH 7.5 and room temperature. In the first experiment, oocytes were exposed to a high concentrated sucrose solution (0.9 M, 1450 mOsm) and the diameter of the oocytes was recorded after 10 min and 1 h. Oocytes were also weighed before and after 1 h of exposure to sucrose. With these data (not shown), we determined that oocytes did not lose more than 25% of their volume or weight, which corresponds to the free water content. For further calculations (see below), the osmotically inactive volume was taken as 75% of the initial oocyte volume. From subsequent experiments, where oocytes were dried in a oven during 24 h, we estimated that the bound water associated with osmotically inactive volume of oocytes is 45% of the total water of the oocyte (data not shown).

### Measurement of water and cryoprotectant permeability of oocytes in hypertonic solutions

The *L*_p _and *P*_EG _of water- and aquaporin-injected oocytes were calculated from their relative volume changes when exposed to 0.9 M sucrose (1460 mOsm) or 1.3 M ethylene glycol (1600 mOsm) in MBS at pH 7.5 for 10 min at room temperature. Within this time period, the compounds were not toxic for the oocytes. The change in oocyte volume were recorded every 10 sec with a video camera as described above. The data were entered into the DIFFCHAM software [70] which allows to estimate, based on the change in concentration of the solutes and the original volume of the oocyte, values of *L*_p_, *P*_EG _and σ for individual oocytes according to the Kedem-Katchalsky model (K-K model) [71] of movement of solutes across cell membranes (see [72] for details). The software was instructed to model for an extracellular step rise in sucrose or ethylene glycol concentration, from 0 to 0.9 M or from 0 to 1.3 M, respectively, with a non-permeating solute of 200 mOsm corresponding to the MBS solution. The partial molar volume of ethylene glycol was 54 cm^3^/mol, and the gas constant (R) was 82.3 cm^3^/atm/mol [31]. The osmotically inactive volume was 75% of the initial oocyte volume as determined above. Five to seven oocytes per construct were analyzed individually for each batch of oocytes. In these experiments, the *P*_f _can be calculated using the formula LpRT/Vw.

### Radioactive ethylene glycol uptake assays

The uptake of [1,2-^3^H]ethylene glycol (American Radiolabelled Chemicals Inc.; 20 Ci/mmol) was determined in oocytes exposed to isotonic and hypertonic solutions at room temperature. Under isotonic conditions, groups of 10 oocytes injected with water or aquaporin cRNA were incubated in 200 μl of isotonic MBS at different pH containing 5 μM (20 μCi) [1,2-^3^H]ethylene glycol, and cold ethylene glycol was added to give 1 mM final concentration. After 1 min (including zero time for subtraction of the signal from externally bound solute), oocytes were washed rapidly in ice-cold MBS three times, and individual oocytes were dissolved for at least 1 h in 400 μl of 10% SDS for scintillation counting.

To determine the ethylene glycol content of oocytes exposed to hypertonic 1.3 M ethylene glycol solution in MBS at pH 7.5, the protocol was the same than above, except that the exposure to the ethylene glycol solution was for 10 min, and the washing was carried out in MBS containing 1.3 M ethylene glycol. An amount of 12 μCi of [1,2-^3^H]ethylene glycol was added to 120 μl of 1.3 M in MBS containing 6 oocytes (resulting in 73000 cpm/μl). The final concentration (in mol/l) of ethylene glycol in each oocyte after 10 min exposure was determined with the formula: [(T_10_- T_0_)/73000] × 1.3 μmol/V; where T_10 _are the cpm in oocytes after 10 min, T_0 _are the cpm in oocytes at time 0 (i.e., oocytes exposed to the solution for one sec), and V is the final oocyte volume (in μl) after 10 min exposure to 1.3 M ethylene glycol, which was estimated from the initial oocyte volume and the changes in volume under 1.3 M ethylene glycol solution previously determined.

### Immunofluorescence microscopy and immunoblotting

For immunohistochemistry, oocytes were fixed 4 h in 4% paraformaldehyde in PBS (137 mM NaCl, 2.7 mM KCl, 100 mM Na_2_HPO_4_, 2 mM KH_2_PO_4_, pH 7.4) and then washed, dehydrated and embedded in paraffin as described [73]. Sections of 7 μm were then rehydrated and blocked in 5% goat serum, 0.1% BSA in PBST (0.1% Tween in PBS). Incubation with a DrAqp3b rabbit affinity-purified antiserum (1:250) was performed overnight at 4°C in 1% goat serum, 0.1% BSA in PBST. The antiserum was raised against a synthetic peptide corresponding to the COOH termini of DrAqp3b (CERLKLSAVSDKDAA) (Agrisera). After washing, sections were incubated with a secondary anti-rabbit IgG FITC-coupled antibody (Sigma) for 2 h. Sections were mounted with fluoromount aqueous anti-fading medium (Sigma). Immunofluorescence was observed and documented with a Zeiss imager.z1 microscope.

For Western blotting, total and plasma membrane protein extracts were isolated from groups of 10 oocytes as described [74]. Protein samples were denaturated at 95°C in Laemmli sample buffer and the equivalent of 1 oocyte (in 15 μl) for total membrane, or 2 oocytes (in 30 μl) for plasma membrane, were deglycosylated by incubation with 500 U of PNGase F (New England Biolabs Inc.) for 3 h at 37°C. The enzyme was inactivated by 5 min incubation at 95°C before electrophoresis of the samples in a 12% SDS-PAGE. Proteins were blotted on Hybond ECL nitrocellulose membrane (Amersham Bioscience), and after blocking in 5% nonfat dry milk in TBST (20 mM Tris, 140 mM NaCl, 0.1% Tween, pH 7.6), membranes were incubated overnight at 4°C with anti-DrAqp3b antibody (1:500) diluted in blocking solution. Bound antibodies were detected with anti rabbit IgG antibody (1:2000) coupled to horseradish peroxidase (Rockland Inc.). Reactive protein bands were detected using Picomax sensitive chemiluminescent HRP substrate (Rockland Inc.).

### Statistics

Data are expressed as mean ± SEM. The data on oocyte water and solute permeability and ethylene glycol content are an average of 2-4 experiments each performed on a different batch of oocytes. Data was statistically analyzed by one-way ANOVA or by the unpaired Student's *t *test; *p *values < 0.05 were considered significant.

## List of abbreviations

AQP: aquaporin; GLP: aquaglyceroporin; *L*_p_: hydraulic conductivity; *P*_f_: osmotic water permeability; *P*_S_: solute permeability; *P*_Gly_: glycerol permeability; *P*_PG_: propylene glycol permeability; *P*_EG_: ethylene glycol permeability; σ: reflection coefficient; MBS: modified Barth's medium; PNGase F: N-Glycosidase F; WT: wild-type.

## Authors' contributions

FC carried out the functional expression in oocytes, site-directed mutagenesis, swelling assays and cryoprotectant uptake experiments, and drafted the first version of the manuscript. FC and JC performed structural analyses and designed the mutant constructs. EL, together with FC and JC, participated in the design of experiments and discussion of the results. JC conceived and coordinated the study, and together with EL wrote the final version of the manuscript. All authors read and approved the final manuscript.

## Supplementary Material

Additional file 1 **Effect of pH on the permeability of control and DrAqp3b-expressing *Xenopus laevis* oocytes determined in solutions with diluted or undiluted ion concentrations**. Osmotic water permeability (*P*_f_; A) and ethylene glycol permeability (*P*_EG_; B) of oocytes expressing 2 ng cRNA of DrAqp3b at different pH. In A, oocytes were preincubated in normal MBS or in isotonic MBS containing 78 mM NaCl and 20 mM sucrose, and subsequently assayed for *P*_f _using 10 times diluted MBS or a hyposmotic bathing solution made by removing the sucrose, respectively. The same values of *P*_f _were obtained by using MBS containing 50 mM or 25 mM NaCl, and 56 mM or 126 mM sucrose, respectively (data not shown). In B, oocytes were preincubated with normal MBS or in isotonic MBS containing 38 mM NaCl and 100 mM sucrose, prior to the swelling assays in isotonic MBS containing 100 mM ethylene glycol. In both A and B, values are the mean ± SEM of a representative experiment (*n* = 6 oocytes). Click here for file

Additional file 2 **Functional characterization of additional DrAqp3b mutants in *Xenopus laevis* oocytes**. Osmotic water permeability (*P*_f_) of oocytes expressing wild-type DrAqp3b (DrAqp3b-WT) or different DrAqp3b mutants at different pH. Values are the mean ± SEM of 2-3 experiments (*n* = 8-10 oocytes per construct). The asterisks indicate significant differences between DrAqp3b-WT and mutants at a given pH (Student's *t* test, *p* < 0.05). Click here for file

Additional file 3 **Forward and reverse primers employed to introduce mutations into the zebrafish Aqp3b cDNA**. The table lists the oligonucleotide primers employed for the site-directed mutagenesis of the zebrafish Aqp3b cDNA. Click here for file
