# Supplementary material for: Design and characterization of genetically engineered zebrafish aquaporin-3 mutants highly permeable to the cryoprotectant ethylene glycol
Source: BMC Biotechnol. 2011 Apr 8;11:34. doi: 10.1186/1472-6750-11-34 (PMC3079631; doi:10.1186/1472-6750-11-34)
Supplement: Additional file 1 — Effect of pH on the permeability of control and DrAqp3b-expressing Xenopus laevis oocytes determined in solutions with diluted or undiluted ion concentrations. Osmotic water permeability (Pf; A) and ethylene glycol permeability (PEG; B) of oocytes expressing 2 ng cRNA of DrAqp3b at different pH. In A, oocytes were preincubated in normal MBS or in isotonic MBS containing 78 mM NaCl and 20 mM sucrose, and subsequently assayed for Pf using 10 times diluted MBS or a hyposmotic bathing solution made by removing the sucrose, respectively. The same values of Pf were obtained by using MBS containing 50 mM or 25 mM NaCl, and 56 mM or 126 mM sucrose, respectively (data not shown). In B, oocytes were preincubated with normal MBS or in isotonic MBS containing 38 mM NaCl and 100 mM sucrose, prior to the swelling assays in isotonic MBS containing 100 mM ethylene glycol. In both A and B, values are the mean ± SEM of a representative experiment (n = 6 oocytes). [file 1472-6750-11-34-S1.PDF]

Additional file 1

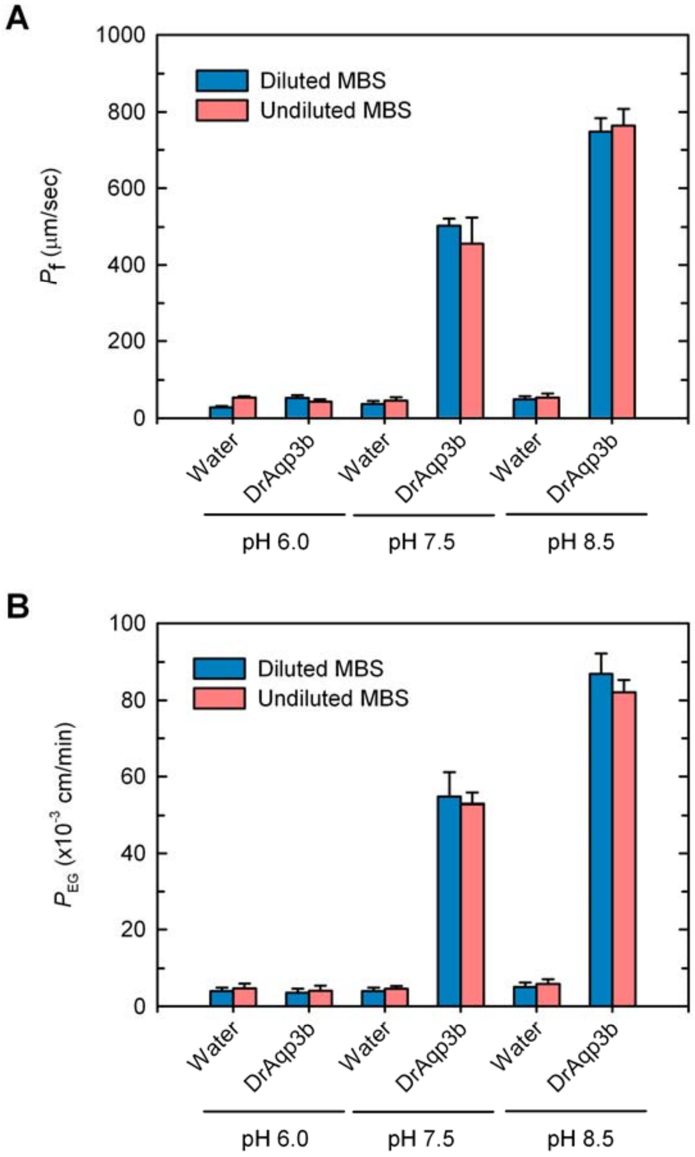

Effect of pH on the permeability of control and DrAqp3b-expressing *Xenopus laevis* oocytes determined in solutions with diluted or undiluted ion concentrations.

Osmotic water permeability (P_f; A) and ethylene glycol permeability (P_{EG}; B) of oocytes expressing 2 ng cRNA of DrAqp3b at different pH. In A, oocytes were preincubated in normal MBS or in isotonic MBS containing 78 mM NaCl and 20 mM sucrose, and subsequently assayed for P_f using 10 times diluted MBS or a hyposmotic bathing solution made by removing the sucrose, respectively. The same values of P_f were obtained by using MBS containing 50 mM or 25 mM NaCl, and 56 mM or 126 mM sucrose, respectively (data not shown). In B, oocytes were preincubated with normal MBS or in isotonic MBS containing 38 mM NaCl and 100 mM sucrose, prior to the swelling assays in isotonic MBS containing 100 mM ethylene glycol. In both A and B, values are the mean ± SEM of a representative experiment (n = 6 oocytes).
