# Supplementary material for: Design and characterization of genetically engineered zebrafish aquaporin-3 mutants highly permeable to the cryoprotectant ethylene glycol
Source: BMC Biotechnol. 2011 Apr 8;11:34. doi: 10.1186/1472-6750-11-34 (PMC3079631; doi:10.1186/1472-6750-11-34)
Supplement: Additional file 2 — Functional characterization of additional DrAqp3b mutants in Xenopus laevis oocytes. Osmotic water permeability (Pf) of oocytes expressing wild-type DrAqp3b (DrAqp3b-WT) or different DrAqp3b mutants at different pH. Values are the mean ± SEM of 2-3 experiments (n = 8-10 oocytes per construct). The asterisks indicate significant differences between DrAqp3b-WT and mutants at a given pH (Student's t test, p < 0.05). [file 1472-6750-11-34-S2.PDF]

Additional file 2

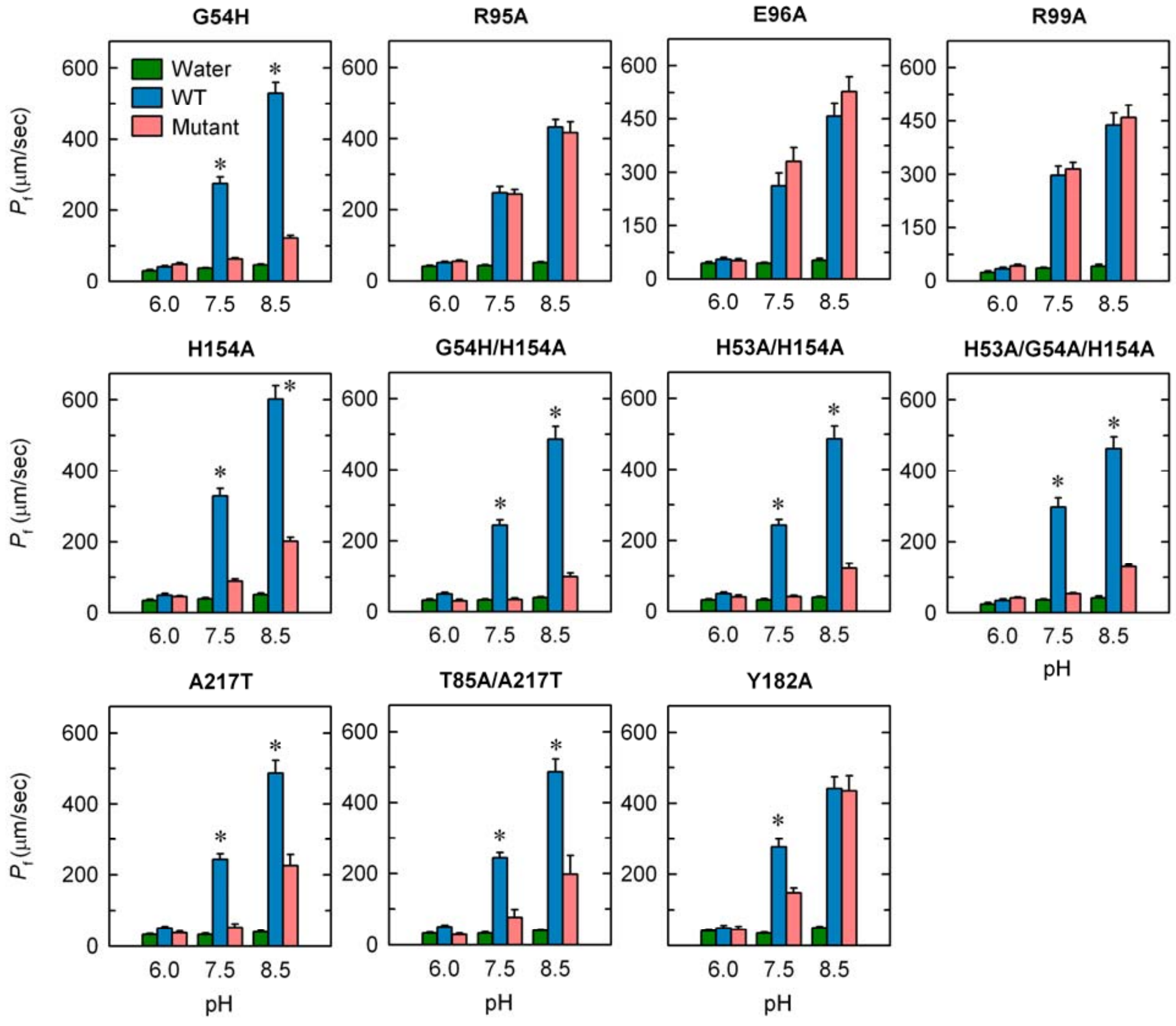

Functional characterization of additional DrAqp3b mutants in *Xenopus laevis* oocytes.
Osmotic water permeability (P_f) of oocytes expressing wild-type DrAqp3b (DrAqp3b-WT) or different DrAqp3b mutants at different pH. Values are the mean ± SEM of 2-3 experiments (n = 8-10 oocytes per construct). The asterisks denote significant differences between WT and mutant DrAqp3b at a given pH (Student's t test, $p < 0.05$).
