# Supplementary material for: Design and characterization of genetically engineered zebrafish aquaporin-3 mutants highly permeable to the cryoprotectant ethylene glycol
Source: BMC Biotechnol. 2011 Apr 8;11:34. doi: 10.1186/1472-6750-11-34 (PMC3079631; doi:10.1186/1472-6750-11-34)
Supplement: Additional file 3 — Forward and reverse primers employed to introduce mutations into the zebrafish Aqp3b cDNA. The table lists the oligonucleotide primers employed for the site-directed mutagenesis of the zebrafish Aqp3b cDNA. [file 1472-6750-11-34-S3.PDF]

Additional file 3

Forward and reverse primers employed to introduce mutations into the zebrafish Aqp3b cDNA

	**Forward primer 5'-3'/Reverse primer 5'-3'**
H53A	GCATATTTTAAGCGGAGGCTCTGCTGGAATGTTTCTGACAG/<br>CTGTCAGAAACATTCCAGCAGAGCCTCCGCTTAAAATATGC
G54H	GCGGAGGCTCTCATCATATGTTTCTGACAGTGAATTTTGC/<br>GCAAAATTCACTGTCAGAAACATATGATGAGAGCCTCCGC
H53A/G54H	GCGGAGGCTCTGCTCATATGTTTCTGACAGTGAATTTTGC/<br>GCAAAATTCACTGTCAGAAACATATGAGCAGAGCCTCCGC
T85A	GGAGGTCACATAAACCCTGCTGTGACCTTTTCTCTCTG/<br>CAGAGAGAAAAGGTCACAGCAGGGTTTATGTGACCTCC
R95A	CTCTGTTTGTTGGGGGCGGAGCCCTGGAGG/<br>CCTCCAGGGCTCCGCCCCCAACAAACAGAG
E96A	CTCTGTTTGTTGGGGAGGGCGCCCTGGAGGAAATTTCCC/<br>GGGAAATTTCCTCCAGGGCGCCCTCCCCAACAAACAGAG
R99A	GGGAGGGAGCCCTGGGCGAAATTTCCCGTTTAC/<br>GTAAACGGGAAATTTCGCCCAGGGCTCCCTCCC
H154A	GCTACGTACCCTTCTAAAGCCCTTACTTTGCTAAATGG/<br>CCATTTAGCAAAGTAAGGGCTTTAGAAGGGTACGTAGC
Y182A	GCCATTGTTGACCCTGCCAATAACCCCATCCCG/<br>CGGGATGGGGTTATTGGCAGGGTCAACAATGGC
A217T	GGCTATGCTGTAAACCCAACCAGAGACTTGGGACCACG/<br>CGTGGTCCCAAGTCTCTGGTTGGGTTTACAGCATAGCC

Mutated codons are underlined.
